# Valorization of Pear Pomace in Taro Gluten-Free Muffins: Composition, Texture, and Sensory Profile

**DOI:** 10.3390/foods14223903

**Published:** 2025-11-14

**Authors:** Dilek Demirbuker Kavak, Betül Aslan Yilmaz, Bilge Akdeniz

**Affiliations:** 1 Food Engineering Department, Engineering Faculty, Afyon Kocatepe University, ANS Campus, 03200 Afyonkarahisar, Türkiye; btlasln32@gmail.com; 2 Food Technology Department, Şuhut Vocational School, Afyon Kocatepe University, Afyon Street, Şuhut, 03800 Afyonkarahisar, Türkiye

**Keywords:** taro, pear pomace, valorization, gluten-free muffin, dietary fiber, total phenolic content, texture profile analysis, principal component analysis

## Abstract

The search for high-quality gluten-free products remains challenging, as they often exhibit poor texture and nutritional deficiencies. The potential of taro (*Colocasia esculenta* L. Schott) flour combined with fruit by-products such as pear pomace (DP) to improve these characteristics remains largely unexplored. It was hypothesized that substituting taro flour with DP would enhance the nutritional profile and sensory quality of gluten-free muffins. Muffins were formulated with taro flour and DP at 0–20% substitution levels. Analyses included flour physicochemical characterization, image-based evaluation of crumb structure, multivariate sensory profiling, and assessment of antioxidant and nutritional properties. Taro flour showed high water-binding capacity, supporting product moisture. At 5% DP, total phenolics and dietary fiber increased by 55% and 32%, respectively, while maintaining control-like texture and porosity. A 10% DP substitution enhanced aroma attributes. Although 20% DP yielded the highest fiber (68%) and phenolics (155%) contents compared to the control, it increased hardness and reduced porosity. Substitution with up to 10% DP effectively balanced nutritional improvement and desirable sensory attributes, demonstrating the valorization potential of pear pomace in taro-based gluten-free muffins.

## 1. Introduction

The valorization of fruit and vegetable by-products is a critical strategy for addressing sustainability challenges and transforming waste into value-added products [1,2,3]. Globally, about 50% of industrially processed fruits and vegetables result in peels, cores, or pomace, which are rich sources of bioactive compounds, dietary fiber, vitamins, and minerals [4]. Incorporating these materials into food products not only enhances nutritional profiles but also mitigates the environmental and economic burdens associated with waste disposal [1,4].

Among fruit processing by-products, pear pomace stands out due to its high dietary fiber and phenolic content [1,5]. In particular, the Deveci pear (*Pyrus communis* L. cv. Deveci) is notable for its juiciness and high levels of beneficial compounds, such as chlorogenic acid, rutin, and other flavonoids with strong antioxidant activity [1,6]. Pomace from common pear varieties has already been incorporated into baked goods such as cereal bars [7], breads [8], and cakes [9]. Given its composition, pear pomace also holds promise as a functional ingredient for enhancing the nutritional profile of gluten-free bakery products. However, the use of Deveci pear pomace remains largely unexplored in gluten-free products. This market is rapidly expanding due to health-driven consumer trends and the growing emphasis on the sustainable valorization of fruit-processing residues [10,11]. This gap is particularly relevant given the rising demand for functional, gluten-free alternatives in bakery products. Yet, commercially available gluten-free products often suffer from lower technological quality and nutritional deficiencies, especially in fiber, protein, and minerals, compared to their wheat-based counterparts [12].

It has been shown that the valorization of fruit and vegetable pomace has been successfully implemented across various baked goods, including gluten-free breads [13,14], muffins [15,16,17], and ready-to-bake snacks [18]. These applications consistently report an increase in nutritional value, dietary fiber content, and antioxidant potential, contributing significantly to the sustainable management of agro-industrial residues [13,15,16,17,18]. However, a primary technological challenge in utilizing high levels of fruit and vegetable by-products is the resulting deterioration of structural quality. The high insoluble fiber content often leads to a reduction in product volume and a significant increase in hardness [15,16,17,18]. These structural challenges can drastically reduce sensory acceptance. The darkening of color and the introduction of undesirable off-flavors at high substitution rates (e.g., typically above 15–20% depending on the pomace type) [13,14] are the main contributing factors, often leading to the rejection of formulations that offer the greatest nutritional benefit.

Taro (*Colocasia esculenta*) flour, a naturally gluten-free tuber, emerges as a promising base ingredient to address these shortcomings. It can enhance nutritional value and impart a natural hue, while maintaining acceptable physical–sensory properties in muffins. This is despite slight reductions in volume and increases in density [19]. Its nutritional richness (e.g., dietary fiber, essential minerals, and amino acids) and distinctive functional properties, including high water absorption and mucilage content, make it particularly suitable for improving texture and nutrient density in gluten-free baked goods [20,21,22]. Although taro flour has been successfully applied in breads and cookies [23], no study has yet strategically employed laboratory-produced taro flour with its distinctive functional properties. This approach could serve as a base to overcome the structural and sensory hurdles commonly encountered when integrating high levels of fruit by-products like pear pomace into gluten-free muffins. This study, through a comprehensive, multidimensional quality characterization, provides the first integrative assessment of this synergistic combination.

To address these challenges, this study strategically selected taro flour and pear (*Pyrus communis* L. cv. Deveci) pomace powder (DP) to create a synergistic combination for gluten-free muffins. Taro flour was produced and used as the gluten-free muffin base for its technological functionality and nutritional capacity, while DP was incorporated as a fibre-, bioactive-, and aromatic-rich valorized by-product. The formulations were prepared at varying DP levels and subjected to comprehensive quality evaluations, including physicochemical composition, antioxidant potential, structural properties (via image processing), texture, and multivariate sensory analysis. This integrative assessment provides new insights into the combined potential of fiber- and bioactive-rich by-products when used with alternative gluten-free bakery formulations, supporting the improvement of functionality and the sustainable valorization of fruit-processing by-products in line with circular economy principles.

## 2. Materials and Methods

### 2.1. Preparation of Taro Flour

The taro (*Colocasia esculenta* L. Schott) corms used in this study were obtained from Becos Farm, a local producer located in Aydıncık, Mersin Province, Türkiye. Taro flour was prepared according to the method described in [24]. Corms were cleaned and manually peeled using stainless-steel knives. The peeled corms were rinsed with water to reduce excessive mucilage and prevent browning, then sliced uniformly into 5 mm thick pieces. The slices were dried in an air oven (Binder 9010-0218, Binder GmbH, Tuttlingen, Germany) at 60 °C for 20 h. The dried slices were ground into flour using a grinder (Demsan, Konya, Türkiye) and sieved through a 250 µm aperture size test sieve (Miprolab, Ankara, Türkiye) to ensure uniform particle size. Finally, the flour was packed in sealed airtight high-density polyethylene (HDPE) bags under dry, light-protected conditions at +4 °C until use.

### 2.2. Proximate Composition Analysis of Taro Flour

The moisture content was determined by drying samples in an oven at 103 °C to a constant mass. Protein content was quantified using the Kjeldahl procedure with a conversion factor of 6.25. Both methods followed established protocols [25]. Ash content was determined gravimetrically. Fat content was analyzed using the Soxhlet extraction method [26], and dietary fiber content was measured according to AOAC (2005) [27]. Finally, carbohydrate content was calculated by difference.

### 2.3. Hydration and Oil Absorption and Color Characteristics of Taro Flour

The hydration and oil absorption properties of taro flour were determined following the method described in [24] with slight modifications. To determine the water binding capacity (WBC), 2 g of taro flour was suspended in 40 mL of distilled water and mixed for 1 h (IKA Werke yellowline RS 10, IKA-Werke GmbH & Co. KG, Staufen, Germany). The suspension was then centrifuged (Hettich Universal 320 R, Hettich GmbH & Co. KG, Tuttlingen, Germany) at 2200 rpm for 10 min. After decanting the supernatant, the wet sample was drained for 10 min and weighed. WBC was calculated as follows in Equation (1):

(1) $$WBC(g/g)=\frac{Weight\;of\;sediment\;after\;centrifugation-Sample\;dry\;weight}{Sample\;weight}$$

Distilled water was added to a centrifuge tube containing the sample. The tube was then incubated at ambient temperature (25 ± 2 °C) for 24 h. After incubation, the supernatant was carefully removed using a dropper, and the remaining sample was weighed. WHC was calculated as follows in Equation (2):

(2) $$WHC(g/g)=\frac{Weight\;of\;sediment\;after\;draining-Sample\;dry\;weight}{Sample\;dry\;weight}$$

To determine the water and oil adsorption capacity (WAC and OAC), 3 g of taro flour was mixed with 30 mL of distilled water or vegetable oil (Olin, Türkiye) in a centrifuge tube, then incubated at 25 ± 2 °C for 1 h. After centrifugation at 2500 rpm for 30 min, the supernatant was removed. The remaining paste was weighed to determine absorption capacity. The absorption capacity was expressed as grams of water or oil absorbed per gram of flour sample on a dry weight basis (g/g).

The color characteristics of taro flour were evaluated with a Chroma Meter CR-400 colorimeter (Minolta Co., Ramsey, NJ, USA). The L* value indicated lightness (ranging from 0 [black] to 100 [white]), while a* represented the green (−a) to red (+a) spectrum, and b* denoted the blue (−b) to yellow (+b) spectrum. The whitening index (WI) of taro flour was calculated using the following Equation (3) [22]:

(3) $$WI=100-\sqrt{{{(100-L}^{*})}^{2}+{\left(a^{*}\right)}^{2}+{\left(b^{*}\right)}^{2}}$$

### 2.4. Thermal Analysis of Taro Flour

Thermogravimetric-differential thermal analysis (TG-DTA) was performed using a Seiko SII TG/DTA 7200 (Seiko Instruments Inc., Chiba, Japan). 10 mg of taro flour was heated from 25 °C to 650 °C at a rate of 10 °C/min under a nitrogen atmosphere (20 mL/min), with 10 mg of α-Al_2_O_3_ used as a reference material. The mass change (TG) and heat flow (DTA) data were recorded simultaneously to evaluate thermal stability and decomposition behavior. A differential scanning calorimeter (DSC) (NETZSCH STA 449 F3 Jupiter, NETZSCH-Gerätebau GmbH, Selb, Germany) was used to analyze the thermal properties of flour under both wet and dry conditions. To determine the intrinsic thermal properties of taro flour, dry analysis was performed by heating the flour sample from 30 °C to 130 °C at a rate of 10 °C/min. To characterize the thermal transitions of hydrated taro flour, samples were prepared at the literature-reported 1:2 (*w*/*w*) ratio [28] and analyzed from 30 °C to 200 °C, covering the temperature range relevant to both starch gelatinization and baking processes. An empty aluminum pan was used as a reference for all DSC measurements.

### 2.5. Particle Size Distribution and Microstructural Characterization of Taro Flour

Particle size distribution measurements were performed on a Malvern Mastersizer 2000 laser diffraction system (Malvern Instruments Ltd., Malvern, Worcestershire, UK) equipped with a wet dispersion unit (Hydro 2000MU). The sample was dispersed in deionized water followed by sonication for 5 min to disperse any agglomerates, following a previously established method [29]. Scanning electron microscope (SEM) micrographs were acquired to examine the granule size and morphology of the flour particles. Images were captured at 500× magnification with a 20 µm scale bar using a LEO Model 440 VP Zeiss microscope (Carl Zeiss AG, Cambridge, UK).

### 2.6. Muffin Preparation

Fresh pears (*Pyrus communis* L. cv. Deveci) were obtained from the Söz retail chain in Afyonkarahisar, Türkiye. Pear pomace was produced using a masticating-type slow fruit juicer (<50 rpm) (Ariston Hotpoint Slow-Juicer PRO, Ariston, İstanbul, Türkiye). Fresh pomace was immediately dried for 20 h at 60 °C in an air oven (Binder 9010-0218, Binder GmbH, Tuttlingen, Germany). Subsequently, the dried pomace was ground using a grinder (Demsan, Konya, Türkiye) and sieved through a 250 µm aperture size test sieve (ISO 3310-1:2000, Miprolab, Ankara, Türkiye). Produced Deveci pear pomace powder (DP) was then stored at 4 °C in airtight high-density polyethylene (HDPE) bags until use.

All muffin formulations included food-grade ingredients, namely: white sugar (Torku, Konya, Türkiye), whole medium eggs (Evrenkaya, Afyonkarahisar, Türkiye), whole cow milk (Torku, Konya, Türkiye), sunflower oil (Orkide, İzmir, Türkiye), baking powder (Dr. Oetker, İzmir, Türkiye), and taro flour.

The gluten-free muffins were prepared using taro flour as the base (control: 100% taro flour) with partial substitutions of 5%, 10%, and 20% Deveci pear pomace powder (DP) of the total flour weight. The 5–20% range was strategically chosen to align with established practices in fruit pomace research [10], allowing us to identify the appropriate level for nutritional and sensory quality while also defining the technological limits of the gluten-free matrix. This range consistently reflects the literature: optimal acceptability for various pomaces (e.g., apple, grape, tomato) typically falls between 5% and 15% [13,14,15,18]. Levels at or above 15–20% enrichment are reported to critically compromise texture and volume in gluten-free matrices [13,15,16,17], thereby establishing the upper boundary for practical application. Based on 100 g of the flour base, the other ingredients were kept constant at: sugar (60 g), eggs (50 g), milk (125 g), sunflower oil (50 g), and baking powder (4 g). Formulations were coded based on the substitution ratio of Deveci pear pomace powder (DP) as follows: C0 (100% taro flour, 0% pomace, control sample), DP5 (95% taro flour, 5% pomace), DP10 (90% taro flour, 10% pomace), and DP20 (80% taro flour, 20% pomace). Initially, the whole egg and sugar were homogenized for 60 s at the lowest speed setting (Level 1) of a hand mixer (Fakir Trinity, Fakir Werke GmbH, Bissendorf, Germany). Sunflower oil, milk, and the flour mixture (containing baking powder, pomace powder, and taro flour) were then added and mixed for 2 min. The batter (30.0 ± 0.2 g) was deposited into paper muffin cups housed in metallic baking trays. Muffins were baked in a preheated oven (Teka HAK 625N, Teka Group, Mönchengladbach, Germany) at 170 °C for 20 min, with separate batches prepared for each formulation. Post-baking, the samples were cooled for 30 min at room temperature before being packaged in airtight plastic bags to retain moisture and stored at a controlled room temperature (22 ± 1 °C).

Each formulation was replicated in two distinct batches, and at least three muffins per batch were subjected to analytical evaluation. All analyses were conducted on the muffins within 48 h of their production. Texture and sensory evaluations were finalized within the initial 24 h to maintain freshness.

### 2.7. Physicochemical Characterization of Muffins

The muffins were evaluated for proximate composition, physical properties, color, and texture. Proximate analysis of muffins was conducted following AOAC methods [27]: moisture (925.09), fat (920.39), protein (954.01), ash (923.03), and dietary fiber (991.43). Available carbohydrates were calculated by difference.

The height of the muffins was determined with a digital caliper. Each muffin was sliced vertically, and the maximum height was recorded as the distance from the base to the apex at the central point. Baking loss and height measurements were taken for twelve muffins per formulation. The weight reduction during baking was computed using the following Equation (4):

(4) $$Baking\;Loss\;(\%)=\frac{\left(Batter\;weight-Muffin\;weight\right)(g)}{Batter\;weight\;(g)}\times 100$$

The crust and crumb color attributes of muffin samples were evaluated with a Chroma Meter CR-400 colorimeter (Minolta Co., Ramsey, NJ, USA). Crumb color was assessed on transversally sliced surfaces, whereas crust color was measured directly on the muffin outer surface. Three measurements per muffin and six muffins per formulation were analyzed. The total color difference (ΔE) for each formulation was calculated against the control sample using Equation (5) described in [30].

(5) $$\Delta E=\sqrt{{{(L}^{*}-{L_{o}}^{*})}^{2}+{\left(a^{*}-{a_{o}}^{*}\right)}^{2}+{\left(b^{*}-{b_{o}}^{*}\right)}^{2}}$$

Texture Profile Analysis (TPA) was conducted on muffin crumb samples using a texture analyzer (TA-XT Plus, Stable Micro Systems, Godalming, Surrey, UK), following a method adapted from [31]. Samples were prepared by cutting muffins horizontally 2.5 cm from the base. A double compression test was applied to 25% deformation using a 36 mm cylindrical aluminum probe at a speed of 1 mm/s, with a 5-s pause between cycles. Analyses were performed within 24 h of baking on three muffins from each of two independent batches. Hardness, springiness, cohesiveness, chewiness, and resilience were determined.

### 2.8. Total Phenolic Content and Antioxidant Activity

The phenolic compounds were extracted from the ground gluten-free muffin samples according to a modified method established by [32]. Three grams of the sample were homogenized with 22.5 mL of a methanol-acetone-water (1:1:1, *v*/*v*/*v*) solution, maintaining a solvent-to-solid ratio of 7.5:1 (mL/g). The extraction was carried out at room temperature (approximately 25 °C) under constant stirring at 600 rpm for 30 min using a magnetic stirrer (RT 15, IKA-Werke GmbH & Co. KG, Staufen, Germany). Following the extraction, the mixture was centrifuged at 6800× *g* for 30 min at 4 °C. The resulting supernatant was collected, filtered, and stored for subsequent analyses. The Total Phenolic Content (TPC) was determined using the Folin–Ciocalteu method as described by Kumar and Sharma [33]. Specifically, 0.5 mL of the extract was mixed with 0.25 mL of Folin–Ciocalteu reagent and 3.75 mL of distilled water. After 3 min, 0.5 mL of a 10% (*w*/*v*) sodium carbonate solution was added. The final mixture was incubated at room temperature for 60 min, and the absorbance was measured at 765 nm against a methanol blank. TPC was calculated using a gallic acid standard curve and expressed as milligrams of gallic acid equivalent per gram of dry sample (mg GAE/g). The antioxidant activity was assessed by measuring the 1,1-diphenyl-2-picrylhydrazyl (DPPH) radical scavenging activity [33]. A 1 mL aliquot of the extract was mixed with 1 mL of a fresh 0.1 mM methanolic DPPH solution. The mixture was vortexed and kept in the dark for 30 min at room temperature. The absorbance was then measured at 517 nm using a spectrophotometer (UV-1800, Shimadzu, Kyoto, Japan) against a methanol blank. The DPPH radical scavenging activity was quantified as a percentage of inhibition (AA) using Equation (6):

(6) $$AA=\left[(A_{b}-A_{s}\right)/A_{b}]\times 100$$

where Ab is the absorbance of the blank and As is the absorbance of the reaction mixture containing the extract.

### 2.9. Quantitative Cellular Structure Characterization by Image Processing

The cellular characteristics of muffin crumbs were evaluated based on the method described in [34]. After cutting the samples horizontally 2.5 cm above the base, high-resolution images (600 dpi) of the crumb morphology were captured using a scanner (Canon e510, Canon Inc., Tokyo, Japan). Each image was then converted to an 8-bit grayscale format and binarized, with white pixels representing a crumb matrix and black pixels corresponding to air cells. Quantitative image analysis was performed using ImageJ (v1.54g, National Institutes of Health, Bethesda, MD, USA) to determine four key structural characteristics: total air cell (void) area (mm^2^), average air cell size (mm), percentage air cell area, and circularity.

### 2.10. Sensory Analysis

Quantitative descriptive analysis was employed to evaluate the sensory characteristics of the muffins, adapting the methodology established in [35]. The panel consisted of ten trained members (*n* = 10) from the Afyon Kocatepe University’s Food Engineering Department, selected based on availability, interest, and prior experience. Panelists received four training sessions (approx. 1 h each) focused on identifying and scaling the relevant attributes. The sensory descriptors were generated and defined during two preliminary consensus sessions. The resulting descriptors, scale anchors, and their standardized definitions are provided as shown in Supplementary Table S1. The protocol for the sensory evaluation was approved by the Afyon Kocatepe University Scientific Research and Publication Ethics Committee for Science and Engineering Sciences (Approval number: 2025/20, Date: 8 September 2025). Written informed consent was obtained from all participants before their involvement in the study. The evaluation was conducted over two separate days to account for day-to-day variation. On each day, each panelist evaluated all four formulations once. The mean sensory score for each formulation was calculated for each of the two evaluation days, and these day means (n = 2 biological replicates per formulation) were used as the experimental unit for statistical analysis. All samples were presented to each panelist in a unique randomized order and were labeled with random three-digit codes to ensure blinding. Palate cleansing using water was enforced between evaluations. Sensory attributes were rated on a 10 cm unstructured scale across five categories: appearance (crumb porosity, crust/crumb color), texture (hardness, springiness), mouthfeel (moistness, oiliness, chewiness), odor (typical/aromatic odor), and taste (typical/aromatic taste, aftertaste). Scale ratings were later quantified for statistical analysis. Multivariate analyses were performed in ClustVis, (custom BETA edition) [36]. Data were preprocessed by row-centering, unit variance scaling, and removal of constant variables. Principal Component Analysis (PCA) was applied using singular value decomposition (SVD) with imputation. Heatmaps were constructed based on correlation distance and average linkage, with dendrograms ordered by the tightest cluster first method and no predefined cluster number.

### 2.11. Statistical Analysis

Data are presented as mean ± standard deviation. For each muffin formulation (C0, DP5, DP10, DP20), two independent production batches were prepared, with a minimum of three muffin samples randomly selected from each batch. The experimental unit for statistical analysis was defined as the mean value calculated from the samples within each independent batch (n = 2 biological replicates per formulation). A one-way analysis of variance (ANOVA) was performed to assess the effect of pear pomace substitution on measured parameters. Data were checked for assumptions of ANOVA. Where ANOVA indicated a significant effect (*p* < 0.05), Duncan’s multiple range test was applied for post hoc pairwise comparisons. The *F*-values and exact *p*-values from the ANOVA are reported for the measured parameters. All statistical analyses were conducted using SPSS v.23 (IBM SPSS Statistics, Armonk, NY, USA).

## 3. Results

### 3.1. Proximate Composition, Hydration, Oil Absorption and Color Properties of Taro Flour

The physicochemical characterization of taro flour confirmed its potential as a gluten-free base ingredient, showing a composition rich in carbohydrates and dietary fiber, coupled with strong water-binding properties (Table 1). Proximate analysis revealed that carbohydrates constituted the major component (69.51%), followed by appreciable amounts of protein, ash, and dietary fiber, whereas moisture and lipid contents were relatively low. Functional property assessments demonstrated a high hydration capacity. The water-binding capacity, water-holding capacity, and swelling power were notably high, while the oil absorption capacity exhibited moderate values. Color parameters indicated high lightness (L* = 81.97) with a yellowish-red hue (a* = 7.50, b* = 10.53) and a whiteness index of 77.81.

### 3.2. Thermal Characteristics of Taro Flour

**Figure 1:**
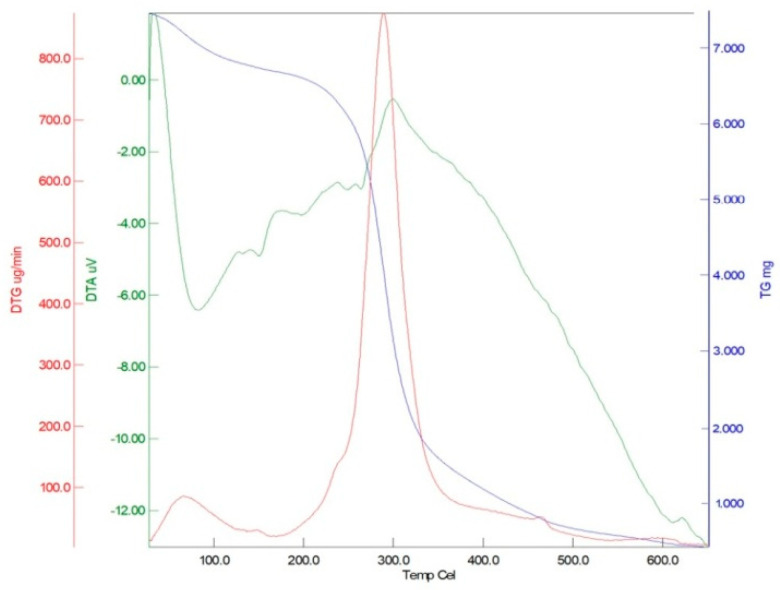
Thermal analysis curves of taro flour: thermogravimetry (TG), derivative thermogravimetry (DTG), and differential thermal analysis (DTA).

The thermal stability and decomposition behavior of taro flour were characterized using thermogravimetric–differential thermal analysis (TG–DTA) and differential scanning calorimetry (DSC). The TG–DTA results revealed a multi-stage degradation pattern (Figure 1). The derivative thermogravimetry (DTG) curve showed a distinct mass loss peak at 73.85 °C, accompanied by a corresponding endothermic signal on the DTA curve at the same temperature. A major exothermic peak was observed on the DTA curve at 289.09 °C, coinciding with a pronounced mass loss step in the DTG curve within the 250–350 °C range. The thermogravimetric (TG) curve stabilized beyond 500 °C, indicating the presence of residual inorganic material. The DSC analysis of hydrated taro flour (1:2, *w*/*w*) did not display a clear gelatinization endotherm (Supplementary Figure S1). Instead, a broad high-temperature endothermic transition was observed, with an onset at 129 °C and a peak at 150.7 °C.

### 3.3. Microstructure and Particle Size Distribution of Taro Flour

**Figure 2:**
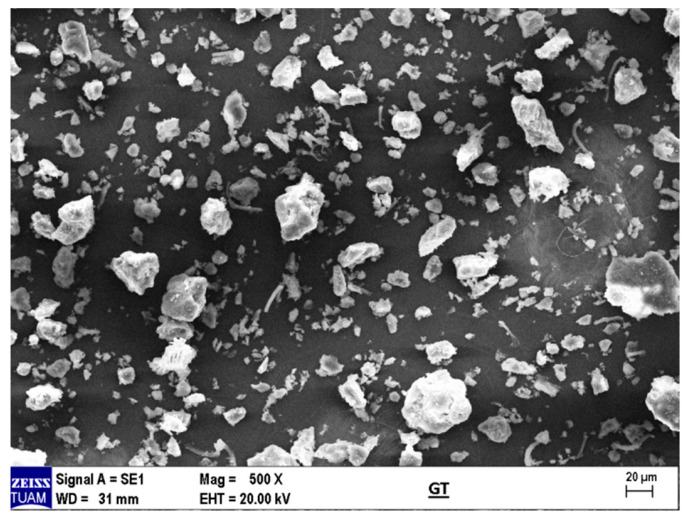
Scanning electron microscopy (SEM) of taro flour.

**Figure 3:**
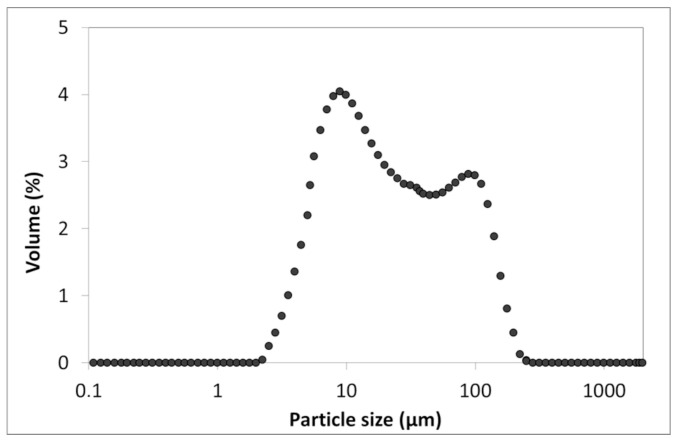
Particle size distribution of taro flour.

Scanning electron microscopy (SEM) analysis of taro flour revealed distinct, small-sized structures with the typical morphology of starch granules, randomly dispersed among larger particles and showing no significant clustering (Figure 2). Particle size distribution (PSD) analysis (Figure 3) demonstrated that taro flour was predominantly composed of fine particles, with a median diameter (D_0.5_) of 19.6 μm. Notably, 90% of the particles (D_0.9_) were smaller than 110 μm, and the span value was approximately 5.3, indicating a moderately broad size distribution containing both fine and coarse fractions.

### 3.4. Proximate Composition, Total Phenolic Content and Antioxidant Activity of Muffins

The incorporation of pear pomace powder (DP) significantly improved the nutritional and bioactive profiles of the gluten-free muffins, primarily through increased dietary fiber and phenolic content, accompanied by a reduction in moisture (Table 2). The most pronounced effects were observed in dietary fiber and moisture content. Dietary fiber exhibited a substantial and concentration-dependent increase, rising by 175% in the DP20 formulation compared to the control (*p* < 0.001). Conversely, moisture content decreased progressively with higher levels of DP substitution, reaching its lowest value in the DP20 sample, which was 15.5% lower than that of the control (*p* < 0.001). Both total phenolic content (TPC) and antioxidant activity (AA) increased significantly with DP incorporation (*p* < 0.001 for both). The TPC of the DP20 muffin was more than 2.5-fold higher than that of the control, confirming the successful transfer of bioactive compounds from the pear pomace into the final product. Similarly, antioxidant activity, measured by DPPH inhibition, was significantly elevated, with the DP20 sample exhibiting a 19.5% higher inhibition rate compared to the control. In contrast, the fat, protein, ash, and carbohydrate contents were not significantly affected by DP substitution (*p* > 0.05), indicating that the core macronutrient composition of the muffins remained largely stable despite the formulation changes.

### 3.5. Color Results of Muffins

Pear pomace substitution significantly affected the color attributes of both the crust and the crumb, with the most pronounced alterations observed in the crust (Table 3). The crust exhibited a distinct color transformation with increasing levels of pomace substitution, becoming progressively darker (decrease in L*), markedly redder (increase in a*), and less yellow (decrease in b*) compared to the control. These shifts resulted in exceptionally high total color difference (ΔE) values, reaching 5.60 for the DP20 sample (*p* < 0.001), indicating a visually perceptible and substantial color deviation. In the crumb, the color changes relative to the control were statistically significant but visually subtle. A clear darkening (decrease in L*) and minor increases in redness and yellowness were observed. The magnitude of the total color change differed markedly between the crust and the crumb. However, all ΔE values for the crumb remained below the visual perception threshold of 3.0 (DP5: 0.40; DP10: 1.01; DP20: 1.37), indicating that these differences were not visually discernible.

### 3.6. Texture, Baking Loss and Height of Muffins

The physical and textural characteristics of gluten-free muffins were markedly affected by the substitution of taro flour with pear pomace powder (DP), showing a significant reduction in product height and a progressive hardening of the crumb, while chewiness remained unexpectedly stable (Table 4). The most prominent physical alteration was the decline in muffin height, which decreased steadily and significantly with increasing levels of pear pomace, reaching a 14.1% reduction in the DP20 sample compared to the control (*p* = 0.002). Conversely, baking weight loss exhibited an upward trend with pomace addition, although the effect did not reach statistical significance (*p* = 0.097).

Textural measurements revealed a clear concentration-dependent response. Hardness measured by TPA using a TA-XT Plus texture analyzer set in “g” force mode, increased systematically with higher DP incorporation, with the DP20 muffins being 28.4% firmer than the control (*p* < 0.001). Similarly, springiness, cohesiveness, and resilience all declined significantly as the substitution level increased (*p* < 0.05 for all). In contrast, chewiness remained remarkably constant across all formulations (*p* = 0.828), suggesting that the perceived chewing effort was maintained despite the pronounced modifications in other textural parameters.

### 3.7. Image Processing Results

**Figure 4:**
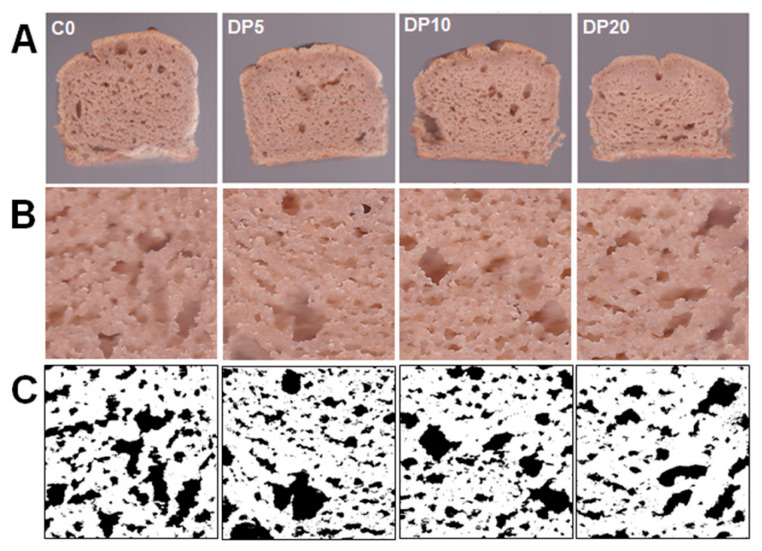
Representative images of muffin crumb morphology and their image processing. (**A**) Scanned cross-sectional image, (**B**) selected region of interest, and (**C**) binarized image for control and pear pomace powder (DP)-substituted muffin samples.

Image analysis demonstrated that the incorporation of pear pomace powder significantly modified the crumb’s pore architecture, resulting in a denser internal matrix while maintaining the average size of individual air cells (Table 5 and Figure 4). The most prominent microstructural alteration was a pronounced reduction in porosity. Both the total air cell area and the percentage of air cell area decreased markedly and consistently with increasing levels of pomace addition (*p* < 0.001). The DP20 muffins exhibited a 24.2% lower total porosity compared to the control, confirming the formation of a much denser crumb structure. This microstructural densification was strongly correlated with the textural properties, as evidenced by a very strong and significant negative correlation between hardness and porosity (air cell area%) (r = −0.932, *p* = 0.001). The resulting simple correlation matrix is provided in Supplementary Table S2. The average air cell size remained statistically unchanged across formulations (*p* = 0.567), indicating that although the number of pores decreased, their fundamental size distribution was largely preserved. Circularity was also significantly influenced by pear pomace addition (*p* = 0.041), showing a non-linear pattern. A moderate substitution (DP5 and DP10) led to increased circularity, reflecting a more uniform pore morphology; however, at the highest substitution level (DP20), circularity declined sharply, suggesting a re-emergence of irregular pore shapes at elevated pomace concentrations.

### 3.8. Sensory Analysis Results

**Figure 5:**
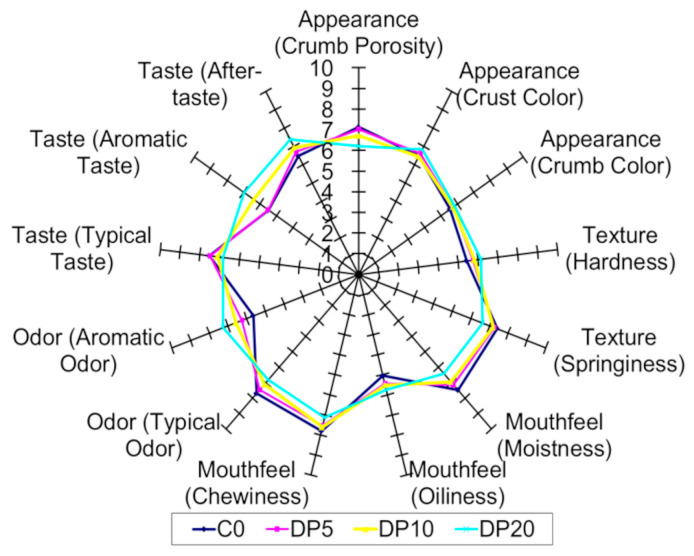
Sensory profile spider web plot derived from Quantitative Descriptive Analysis (QDA) of muffin samples with different pear pomace powder (DP) concentrations.

The sensory profiles of the muffins, evaluated via Quantitative Descriptive Analysis (QDA), revealed significant and concentration-dependent alterations in response to dried pear pomace (DP) substitution, as visually represented in Figure 5. Results demonstrated a concentration-dependent decrease in crumb porosity (*F* = 16.33, *p* = 0.01), with a 13.1% reduction at 20% DP. Crust color intensity increased at higher DP levels, whereas changes in crumb color with DP substitution remained minimal (<5.0%) and not significant (*F* = 2.53, *p* = 0.195). Hardness exhibited a significant, concentration-dependent increase (*F* = 19.05, *p* = 0.008), with the 20% DP substitution showing a 13.2% higher value compared to the control. Conversely, springiness demonstrated a significant and concentration-dependent decrease (*F* = 46.33, *p* = 0.001), with a 10.5% reduction at the 20% DP level. These substantial textural changes were primarily driven by the highest substitution level, as moderate levels (5–10% DP) did not significantly alter hardness or springiness compared to the control. Mouthfeel was influenced by DP addition; at 20% DP, moistness decreased by 14.3% and oiliness increased by 14.1%. Chewiness decreased by 8.5% at the highest substitution but was unaffected at lower levels. Flavor attributes showed a progressive decrease in typical odor (*F* = 49.00, *p* = 0.001) and typical taste (*F* = 40.00, *p* = 0.002) with increasing DP, whereas aromatic odor (*F* = 177.00, *p* = 0.000) and taste (*F* = 201.00, *p* = 0.000) increased markedly, particularly at 10–20% DP. Aftertaste also improved consistently.

**Figure 6:**
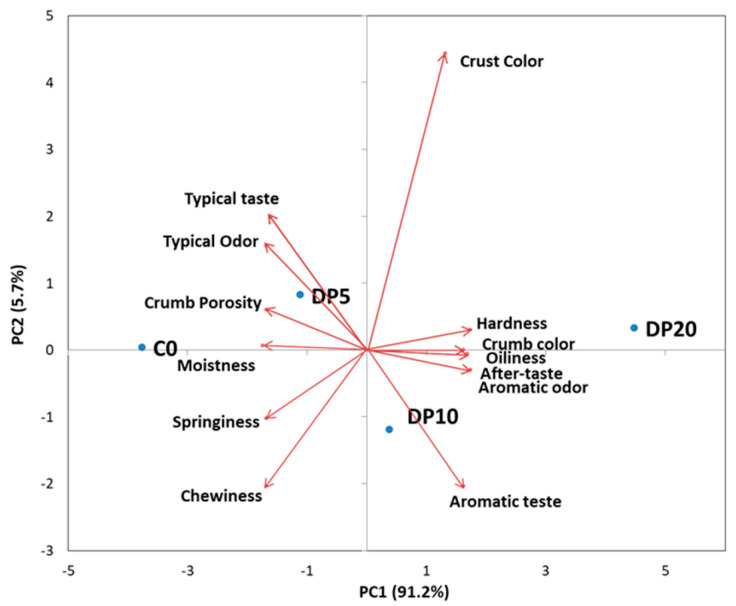
Principal component analysis (PCA) biplot of Quantitative Descriptive Analysis (QDA) sensory profiles of muffin samples.

**Figure 7:**
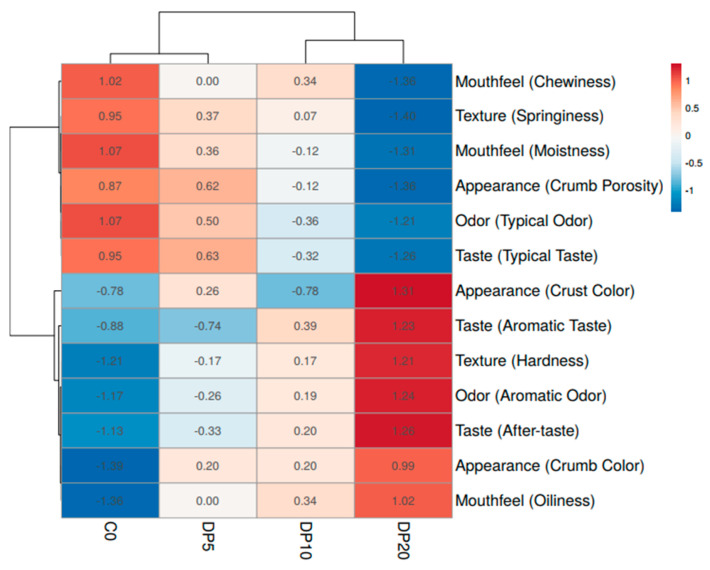
Heatmap of sensory attributes for control and pear pomace powder (DP)-substituted muffin samples based on Quantitative Descriptive Analysis (QDA).

Principal Component Analysis (PCA) (Figure 6) explained 91.2% of the variance on PC1 and 5.7% on PC2. The component loadings are provided in Supplementary Table S3. C0 and DP5 clustered closely, indicating similarity in sensory profiles, while DP20 was separated along PC1, associated with higher hardness, oiliness, aromatic attributes, and lower porosity, moistness, and springiness. DP10 occupied an intermediate position. The heatmap (Figure 7) corroborated these findings. C0 and DP5 formed a single cluster, while DP20 showed high positive z-scores for hardness, oiliness, aromatic odor/taste, and aftertaste, and negative z-scores for porosity, springiness, moistness, and typical odor/taste.

## 4. Discussion

### 4.1. Functional, Physicochemical, and Thermal Properties of Taro Flour

The proximate composition of the taro flour is presented in Table 1. The protein content was determined to be consistent with the values reported for various taro cultivars in previous studies [22,37]. This protein content may be influenced by mucilages, which are rich in proteins [38], thereby affecting the batter’s viscoelastic properties. The moisture content was found to be low, a characteristic that enhances storage stability by inhibiting microbial activity [22]. Our measured value aligns with the typical moisture range (9–12.22%) reported for taro flour [39]. Ash content, serving as an indicator of mineral content, was measured and found to be consistent with values reported for most tropical tubers [22]. Lipids were among the least abundant compounds, consistent with the general understanding of taro flour as a low-fat food source [26]. For comparative purposes, crude lipid content in various taro flour cultivars has been documented as 2.30 ± 0.08% for Altoran and approximately 0.96–0.99% for Josaengjong and Jaeraejong [37]. As expected, total carbohydrates constituted the major component of the composition, which is expected as taro tubers are well-known for their high carbohydrate content, primarily comprising starch [39]. The dietary fiber content, crucial for both flour functionality and human health, was determined. A high dietary fiber content, particularly incorporating resistant starch, is associated with delayed digestion and a reduced prevalence of obesity and diabetes [39]. Our gluten-free muffins thus demonstrate a nutritional advantage over those made with refined white wheat flour, which typically provides around 4% fiber [40]. This suggests that the higher fiber content in taro-based products may contribute to improved glycemic control and enhanced satiety effects compared to conventional wheat flour alternatives.

The measured hydration properties of taro flour, including Water Binding Capacity (WBC), Water Holding Capacity (WHC), and Swelling Power (SP), align with values reported in the literature [37]. This suggests an intact starch granule structure. The Oil Absorption Capacity (OAC) of the flour indicates moderate lipid interaction. The flour’s fiber content notably influences these functional properties, as previous studies have consistently shown that a higher dietary fiber content enhances both water and oil absorption [37,41]. This high water-binding capacity of taro flour offers significant advantages for its integration into muffin formulations. These inherent properties are conducive to achieving a smoother texture and enhanced moisture retention in the final product. These properties can also contribute to improved batter stability [42].

Regarding color parameters, the L* value falls within the lower-to-mid range of reported taro flours (L*: 81–94) [39], indicating a slightly darker tone. This reddish shift can be attributed primarily to enzymatic browning during flour preparation, though heat-induced amino carbonyl reactions during baking may have also contributed. While the b* value aligns with common yellowish hues (3.0–13.8), all taro flours typically exhibit positive b* values, indicating yellowness [43]. The Whiteness Index (WI) value is slightly lower than the typical range reported for white taro flours (WI: 83–93) [22], but aligns well with values observed in yellow taro flours (WI: 67.12–78.75) [44]. This intermediate whiteness level reflects the combined influence of reduced brightness (L) and more pronounced chromatic tones (a and b). Generally, lower L* (brightness) and higher a* (redness) values result in reduced WI. These observed color variations may arise from processing conditions (e.g., drying temperature/time) or varietal differences. Additionally, enzymatic browning, often mediated by polyphenol oxidase [43], can further influence color parameters, as taro flour’s appearance is known to be sensitive to both drying methods and cultivar selection.

The thermal analysis of the taro flour provided critical insights into its stability and behavior. A distinct exothermic peak on the DTA curve at coincided with a significant mass loss peak on the DTG curve, marking the primary degradation stage. This decomposition behavior, a key indicator of the material’s thermal instability, is consistent with literature reports for similar starch-based systems [45,46,47]. The TGA results indicated a significant mass loss between 250 and 350 °C, mainly due to the thermal decomposition of starch and fibrous components under inert conditions. This observed weight loss is reported to strongly correlate with the starch content of flours [48], thereby reinforcing the use of this thermal degradation process as a key indicator of the flour’s thermal stability. The weight loss pattern is consistent with observations in other gluten-free flours (e.g., buckwheat) that undergo similar thermal decomposition within comparable temperature ranges (266–320 °C) [45]. The stabilization of the curve beyond 500 °C indicates the final inorganic ash content [47,48]. Regarding starch transitions, the DSC analysis of hydrated taro flour (Supplementary Figure S1) did not exhibit a distinct gelatinization endotherm. This absence is attributed to methodological constraints. The unsealed pans permitted water evaporation during heating, thereby limiting the accuracy of the gelatinization assessment. Therefore, these DSC data are considered qualitative. Instead, a broad high-temperature endothermic transition was observed, reflecting the hydrothermal degradation of starch components [48], possibly due to residual moisture. From a practical perspective, the onset of major degradation occurs above 250 °C. This is well beyond conventional baking temperatures (180–200 °C), demonstrating that taro flour possesses substantial thermal stability. This property ensures that its starch–fiber matrix remains structurally intact during thermal processing, thereby contributing to crumb stability in gluten-free muffin formulations. The high thermal onset observed in TGA indirectly supports the resilience of taro flour during baking. For a conclusive analysis of its gelatinization behavior, future studies employing sealed-pan DSC protocols are recommended to distinguish water loss from true phase transitions.

The microstructural and granulation properties of the produced taro flour provide a mechanistic explanation for its observed functional behavior. These properties include its high water-binding capacity and its suitability for use in gluten-free muffin systems. The SEM analysis revealed distinct, small-sized starch granules randomly dispersed among larger particles, with no significant clustering (Figure 2). The granules, typically ranging between 1–20 μm, reflect the starch-rich nature of taro flour and are consistent with literature reports [42]. This specific morphology and size distribution, which is influenced by botanical origin [42], directly governs functional properties. In muffin formulations, such fine starch granules contribute to uniform gel formation, improved texture, and reduced brittleness by enhancing water absorption and binding capacity [29,42].

Particle size distribution analysis revealed that the taro flour exhibited a profile dominated by fine particles (Figure 3). The prevalence of these fine particles facilitates uniform hydration and gel formation in the muffin batter. The span value indicated a moderately broad distribution, confirming the presence of a mixture of very fine and some coarser particles. This granulation profile is advantageous for gluten-free systems, as the fine particles enhance binding capacity and reduce brittleness, while the coarser fraction may contribute to structure. The observed size range is consistent with established literature values (e.g., 1.067–64.19 µm as reported in [29]) and characteristic properties of taro flour, where over 60% of particles typically fall below 100 µm [25]. Overall, this particle size distribution helps minimize over-packing of the starch matrix while supporting the desired texture [26].

### 4.2. Physicochemical, Bioactive, Textural and Cellular Structure of Muffins

The significant changes in the proximate composition and bioactive properties of the muffins, as presented in Table 2, directly resulted from the partial substitution of taro flour with pear pomace powder (DP). This substitution yielded a nutritionally enriched and functionally fortified product. A substantial increase in dietary fiber content in the DP20 sample reflected the inherent fiber richness of pear pomace [5]. The incorporation of such by-products aligns with current strategies to increase the dietary fiber content of gluten-free products, which are often deficient in this component [12]. This improvement is not only nutritionally beneficial, as it may be associated with potential health effects such as promoting satiety and contributing to better glycemic control [49,50] but also technologically relevant. The high fiber content directly influenced other muffin properties, including reduced moisture and increased hardness observed in this study. The progressive decrease in moisture content with increasing DP substitution can be attributed to compositional differences between the two ingredients. While taro flour possesses notable water-binding capacity, its starch fraction plays a crucial role in water retention through gelatinization during baking [51]. Pear pomace exhibits a different hydration mechanism and lower starch content. This substitution likely reduced the overall water-holding capacity of the muffin matrix, resulting in a drier crumb. The stability of fat, protein, and carbohydrate contents across formulations indicates that the core macronutrient matrix of the muffins remained largely unaffected. Thus, DP substitution primarily enriched dietary fiber and phytochemicals, potentially enhancing functional properties without significantly altering the basic nutritional composition.

The marked enhancement in total phenolic content (TPC) and antioxidant activity (AA) underscores the success of the valorization approach. The increase in TPC confirms the efficient transfer of polyphenols from pear pomace into the muffin matrix. Pear pomace is a concentrated source of bioactive compounds, including procyanidins, hydroxycinnamic acids (e.g., chlorogenic acid), and flavonoid glycosides such as rutin [1,52]. Notably, the control sample containing only taro flour exhibited baseline antioxidant activity, which is consistent with the presence of intrinsic antioxidants such as flavonoids and carotenoids in taro [20]. The further significant increase in AA with DP addition highlights the synergistic potential of combining taro flour with fruit-derived by-products. The observed antioxidant capacity may be attributed not only to the phenolic compounds of pear pomace but also to the potential formation of Maillard reaction products during baking, which are known to exhibit antioxidant properties [39,44,53]. It should be noted that the DPPH assay employed in this study is sensitive to both phenolic compounds and Maillard reaction products. Therefore, future studies using the ABTS assay, which allows better differentiation of these contributions, would be valuable for further elucidating the sources of antioxidant activity.

The color of baked goods is a critical quality attribute, influencing consumer perception and acceptability [1]. In this study, pronounced color changes were observed in the crust, as shown in Table 3. DP substitution altered the color parameters of both the crust and crumb, with the most substantial changes occurring in the crust. In the crust, DP substitution caused a marked color change, as indicated by the total color difference values. The significant increase in redness and decrease in yellowness led to a darkening and more reddish-brown crust. The ΔE values far exceeded the visual perception threshold (ΔE > 3), confirming a visually striking color change. This color development is primarily due to heat-induced non-enzymatic reactions in the crust, namely the Maillard reaction and caramelization, which are pronounced at the high temperatures reached by the crust during baking [2,9]. The high F-values for crust parameters, particularly for b* and ΔE, underscore the powerful and consistent impact of pear pomace on the product’s external appearance. In the crumb, the color changes were more subtle but still statistically significant. The observed darkening and slight increase in redness and yellowness are attributed to the native color of the taro flour and the pear pomace powder [6] rather than extensive thermal reactions, as the crumb does not reach the same high temperatures as the crust. The fact that all crumb ΔE values remained below the visual perception threshold of 3.0 indicates that the internal color differences, while measurable, were not readily discernible to the human eye [35]. This objective color measurement aligns with the sensory evaluation. Panelists did not report a noticeable difference in crumb color compared to the control, confirming that the visual appeal was maintained.

The physical and textural properties of the muffins were systematically influenced by the level of pear pomace substitution, as detailed in Table 4, revealing clear trends in moisture loss, product structure, and texture. The progressive rise in weight loss may be attributed to altered water dynamics due to the high dietary fiber content of DP. Concurrently, the significant decrease in height suggests that DP substitution may interfere with the batter’s ability to incorporate and retain air, ultimately leading to a denser product structure. This effect is commonly observed when fiber-rich ingredients are incorporated, as high fiber content can disrupt gas retention, reducing volume and increasing density [2,54,55]. In gluten-free systems, the absence of a gluten-derived structural network makes the added fiber’s role even more critical, working alongside taro flour’s starch and protein to determine the final product’s structure and volume [39,54]. The textural properties were profoundly and systematically affected. These opposing trends highlight a key technological trade-off in utilizing fruit pomace at higher concentrations as a functional ingredient. Hardness increased strongly and systematically with higher DP levels, consistent with typical fiber enrichment effects [56]. Springiness, cohesiveness, and resilience decreased, reflecting a firmer, less elastic, and more crumbly matrix. A critical threshold was observed at 20% substitution, where the pronounced fiber content led to a significant increase in hardness, consistent with reports that high pomace levels disrupt protein networks and reduce air incorporation [10,12,57]. The practical implication of this instrumental hardening was also reflected in sensory evaluation, where the 20% DP muffins were rated as significantly harder and less springy than the control. In contrast, chewiness remained remarkably consistent across all treatments, suggesting a complex interaction where increased hardness was counterbalanced by reduced springiness and cohesiveness. This maintained a similar overall chewing effort, which is important for consumer acceptability [57]. This is likely due to the specific water-binding capacity of the DP and taro flour, which maintained crumb moisture and plasticity, preventing brittleness [54]. These results suggest that 5–10% DP substitution effectively enhances nutrition with minimal textural impact. This finding is supported by sensory data, where these samples showed no significant textural deviations from the control, whereas 20% substitution introduces significant hardening. This defining threshold limits the application of high DP concentrations to specialized high-fiber products, where texture is a secondary consideration.

The cellular structure of the muffin crumbs, quantified through image analysis, provides a direct physical explanation for the observed textural properties (Table 5). In gluten-free systems, the absence of a gluten network inherently challenges gas retention [11,58]. The incorporation of DP further modifies aeration. Its high fiber content competes with starch and protein for water, altering batter rheology and disrupting air cell stability [11,59]. The visual evidence from image analysis (Figure 4) clearly shows a reduction in crumb porosity as DP substitution increases. This reduction explains the corresponding increase in hardness at higher substitution levels. The stability of average air cell size across all samples provides a mechanistic explanation for the consistent chewiness observed in TPA. Even though the matrix becomes denser at higher DP levels, the fundamental air cell units that maintain elasticity are preserved. A 5–10% DP substitution resulted in improved circularity of air cells, corresponding to minimal increases in hardness (≈12% at 10% DP) and high springiness retention (over 90% of control). These results indicate that DP fibers interact effectively with the taro flour matrix, stabilizing gas cells and supporting textural integrity. This preserved structural integrity at low-to-moderate substitution levels underpins the positive sensory ratings for texture. In contrast, 20% DP substitution led to significantly reduced porosity and increased hardness, illustrating a direct structure–texture relationship: higher fiber concentrations limit air entrapment, producing a denser and firmer crumb—a principle well-documented in baking science [56]. Microstructural analysis confirms that DP modifies muffin structural integrity in a concentration-dependent way. The 5–10% DP range maintains a uniform, circular pore structure compatible with desirable texture. In contrast, 20% DP leads to a denser matrix with reduced porosity. It is important to acknowledge the inherent methodological considerations in image-based microstructure analysis. The binarization process, which relies on thresholding to distinguish air cells from the crumb matrix, can introduce a degree of subjectivity. In addition, variations in the cutting plane of the muffin sample may affect the observed pore morphology and size distribution. Therefore, the absolute values of the microstructural parameters should be interpreted considering this variability. However, the primary objective of this analysis was not to define universal benchmarks. Instead, it aimed to provide a controlled comparative assessment of the relative differences between the control and DP-substituted samples, all prepared and analyzed under identical conditions. The strong and consistent trends observed across replicates—such as the parallel increase in instrumental hardness with a decrease in total air cell area—demonstrate internal consistency. This confirms that the method provided a robust and reproducible basis for comparative evaluation.

### 4.3. Sensory Evaluation

Quantitative Descriptive Analysis (QDA) and multivariate analyses indicate that DP substitution produces concentration-dependent sensory modifications. The reduction in crumb porosity and increase in crust color at higher DP levels are consistent with the impact of fiber-rich ingredients on air cell stability and matrix structure during baking [59,60]. The increased hardness and reduced springiness observed at 20% DP reflect a firmer, less elastic matrix, whereas moderate DP levels (5–10%) preserve textural properties comparable to the control. Mouthfeel changes, including reduced moistness and increased oiliness at 20% DP, are attributed to the high water-binding capacity of dietary fibers affecting water and fat distribution in the matrix [59]. Flavor modifications—diminished typical odor and taste but enhanced aromatic notes—are associated with the transfer of volatile compounds from pear pomace [11,55]. The PCA biplot (Figure 6) and heatmap (Figure 7) clearly differentiate the samples based on DP concentration, highlighting the concentration-dependent sensory trade-off. The close similarity between the control (C0) and the 5% DP sample demonstrates that nutritional enhancement can be achieved without compromising the core sensory identity of the product. In contrast, the 20% DP sample forms a distinct cluster, characterized by significant textural alterations and strong aromatic notes, defining it as a high-fiber, fruit-forward product for a niche market. The 10% DP sample represents a pivotal intermediate, where the aromatic benefits of the pomace become clearly evident, but textural compromises due to fiber-rich pomace content begin to emerge. Collectively, the results demonstrate that a 5–10% DP substitution level provides the most viable range, balancing nutritional enrichment with acceptable sensory quality for broader consumer acceptance.

### 4.4. Sustainability, Scale-Up Considerations, and Future Perspectives

The formulation developed in this study valorizes a food-grade by-product (pear pomace) using water-only processing and low-temperature dehydration. This approach avoids organic solvents and their associated environmental and recovery burdens. To ensure the long-term applicability of this approach and facilitate its industrial adoption within a circular economy framework, the following considerations should be highlighted: (i) Energy Efficiency in Drying: The specific energy consumption for drying both taro slices and pear pomace should be quantified in future work, specifically the energy required for drying at 60 °C for 20 h. Future studies should explore energy reduction strategies, such as exhaust air heat recovery or optimized convective drying parameters, which would be beneficial for improving the overall sustainability of the process. (ii) Raw Material Standardization: Standardizing the milling and sieving of pear pomace to a consistent particle size (e.g., less than 250 µm) is recommended to ensure batch-to-batch consistency in functional properties like water binding and its impact on the final product’s structure. (iii) Shelf-Life and Packaging: Selecting packaging with appropriate moisture and oxygen barrier properties is key to maintaining product quality. A practical shelf-life assessment is suggested for commercial translation, comparing key formulations (e.g., control, DP5, and DP10) stored in moisture/oxygen-barrier films under both ambient (e.g., 20–22 °C) and accelerated (e.g., 30 °C) conditions. The study should monitor textural changes via Texture Profile Analysis (hardness, springiness), moisture migration (water activity, mass loss), color stability, and microbiological safety (total aerobic count, yeasts/molds). Monitoring textural changes, moisture content, color stability, and microbiological safety over time under defined storage conditions would help establish practical shelf-life and understand the impact of pomace substitution on product stability. Additionally, benchmarking these formulations against leading commercial gluten-free products through a consumer acceptance study is essential to validate their market potential. (iv) Holistic Sustainability Assessment: Conducting a concise Life Cycle Assessment focusing on key inputs like dryer energy consumption and packaging could help quantify the circular-economy benefits of the proposed valorization pathway, thereby supporting sustainable product development and industrial scalability.

## 5. Conclusions

This study demonstrated that pear pomace powder (DP) can be effectively integrated into taro-based gluten-free muffins, not only for by-product valorization. This integration also enhanced their nutritional and sensory quality. Characterization of taro flour highlighted its high water-binding capacity, which supports a moist and tender texture in gluten-free muffins. Its combination with DP (DP) further enhanced the formulation by increasing dietary fiber and polyphenol content and imparting distinctive aromatic attributes. Moderate DP levels (5–10%) achieved the most favorable balance, significantly enhancing bioactive properties (e.g., phenolics increased by 55% and fiber by 32% at 5% DP) and aromatic flavor while preserving textural integrity and microstructure. Comprehensive sensory analysis supported by PCA biplot and heatmap confirmed these concentration-dependent effects: 5% DP closely matched the control with enhanced aroma, 10% DP showed an intermediate sensory profile, and 20% DP diverged with firmer texture and stronger fruity notes. However, a clear nutrition–texture–sensory trade-off was observed at higher substitution levels. For instance, 20% DP maximized fiber and antioxidant activity but also resulted in a denser crumb, higher hardness, and perceptible shifts in sensory quality. By coupling image processing with sensory profiling, this study provides methodological insight into how fiber-rich bioactive by-products interact with alternative gluten-free flours. The results support the suitability of taro flour as a gluten-free base that, when partially substituted with Deveci pear pomace, enables the development of nutrient-dense and consumer-acceptable functional bakery products while promoting the sustainable valorization of fruit-processing by-products. Building on these findings, future studies could further optimize shelf-life by refining criteria for textural integrity, moisture retention, color stability, and microbial safety. These improvements provide practical guidance for commercial adoption and ensure product stability in formulations where pear pomace partially substitutes taro flour.

## Figures and Tables

**Table 1:** Proximate composition, hydration, oil absorption, and color parameters.

Property	Parameter	Value
Proximate composition (%)	Moisture content	9.63 ± 0.32
	Fat content	0.52 ± 0.04
	Protein content	8.70 ± 0.62
	Ash content	6.49 ± 0.24
	Fiber content	5.16 ± 0.09
	Carbohydrates (by difference)	69.51 ± 0.56
Hydration and Oil Absorption Properties	Water binding capacity (g/g)	3.81 ± 0.32
	Water holding capacity (g/g)	3.19 ± 0.16
	Water adsorption capacity (g/g)	3.05 ± 0.11
	Oil adsorption capacity (g/g)	1.86± 0.09
Color parameters	L* (lightness)	81.97 ± 1.15
	a* (redness/greenness)	7.50 ± 0.08
	b* (yellowness/blueness)	10.53 ± 0.45
	WI (whiteness index)	77.81 ± 1.02

**Table 2:** Proximate composition, total phenolic content (TPC), and antioxidant activity (AA) of control and Deveci pear pomace powder (DP)-substituted muffin samples.

Sample	Moisture	Fat	Protein	Ash	Fiber	CHO	TPC	AA
C0	19.48 ± 0.2 ^d^	20.82 ± 0.18 ^b^	7.98 ± 0.20 ^a^	3.41 ± 0.08 ^a^	1.77 ± 0.1 ^a^	46.54 ± 0.01 ^a^	1.65 ± 0.08 ^a^	56.80 ± 0.26 ^a^
DP5	18.61 ± 0.31 ^c^	20.64 ± 0.23 ^ab^	7.95 ± 0.14 ^a^	3.56 ± 0.06 ^ab^	2.55 ± 0.07 ^b^	46.69 ± 0.55 ^a^	2.16 ± 0.07 ^b^	59.09 ± 0.18 ^b^
DP10	17.54 ± 0.21 ^b^	20.46 ± 0.20 ^ab^	7.92 ± 0.11 ^a^	3.5 ± 0.07 ^ab^	3.33 ± 0.11 ^c^	47.25 ± 0.34 ^a^	2.99 ± 0.10 ^c^	62.42 ± 0.16 ^c^
DP20	16.47 ± 0.25 ^a^	20.24 ± 0.16 ^a^	7.88 ± 0.10 ^a^	3.61 ± 0.04 ^b^	4.87 ± 0.1 ^d^	46.93 ± 0.37 ^a^	4.21 ± 0.12 ^d^	67.90 ± 0.26 ^d^
*F*-value	55.47	3.321	0.178	3.442	374.59	2.522	282.81	966.32
*p*-value	<0.001	0.138	0.906	0.132	<0.001	0.196	<0.001	<0.001

Different superscript letters (^a–d^) within the same column indicate significant differences according to Duncan’s multiple range test (*p* < 0.05).

**Table 3:** Color parameters of control and pear pomace powder (DP)-substituted muffin samples.

Sample	Crust L*	Crust a*	Crust b*	Crust ΔE
Crust				
Control	58.38 ± 0.33 ^c^	11.97 ± 0.08 ^a^	33.24 ± 0.14 ^d^	
DP5	57.40 ± 0.01 ^b^	12.22 ± 0.21 ^a^	30.49 ± 0.11 ^c^	2.85 ± 0.11 ^a^
DP10	57.16 ± 0.17 ^ab^	13.82 ± 0.04 ^b^	28.99 ± 0.09 ^b^	4.80 ± 0.02 ^b^
DP20	57.04 ± 0.21 ^a^	13.95 ± 0.09 ^b^	28.18 ± 0.08 ^a^	5.60 ± 0.09 ^c^
*F-*value	16.813	142.052	868.364	19,996.47
*p-*value	0.010	<0.001	<0.001	<0.001
Crumb				
Control	64.27 ± 0.11 ^d^	9.98 ± 0.01 ^a^	17.05 ± 0.06 ^a^	
DP5	63.89 ± 0.06 ^c^	10.01 ± 0.04 ^a^	17.18 ± 0.04 ^ab^	0.40 ± 0.04 ^a^
DP10	63.33 ± 0.14 ^b^	10.24 ± 0.01 ^b^	17.30 ± 0.05 ^bc^	1.01 ± 0.12 ^b^
DP20	62.98 ± 0.07 ^a^	10.30 ± 0.08 ^b^	17.37 ± 0.04 ^c^	1.37 ± 0.09 ^c^
*F-*value	64.330	26.823	17.570	1859.99
*p-*value	0.001	0.004	0.009	<0.001

Different superscript letters (^a–d^) within the same column indicate significant differences according to Duncan’s multiple range test (*p* < 0.05).

**Table 4:** Weight loss, height, and texture profile analysis of control and pear pomace powder (DP)-substituted muffin samples.

Sample	Weight Loss (%)	Height (cm)	Hardness (g)	Springiness	Cohesiveness	Chewiness (g)	Resilience
C0	16.70 ± 0.66 ^a^	4.05 ± 0.07 ^c^	280.04 ± 2.98 ^a^	0.74 ± 0.02 ^c^	0.908 ± 0.01 ^c^	188.15 ± 10.11 ^a^	0.595 ± 0.01 ^d^
DP5	17.43 ± 0.52 ^ab^	3.88 ± 0.04 ^b^	284.55 ± 2.62 ^a^	0.718 ± 0.01 ^bc^	0.882 ± 0.02 ^bc^	180.17 ± 0.97 ^a^	0.563 ± 0.01 ^c^
DP10	18.15 ± 0.68 ^ab^	3.80 ± 0.07 ^b^	314.02 ± 3.96 ^b^	0.692 ± 0.01 ^b^	0.850 ± 0.01 ^ab^	184.75 ± 7.19 ^a^	0.536 ± 0.01 ^b^
DP20	18.80 ± 0.61 ^b^	3.48 ± 0.04 ^a^	359.50 ± 4.74 ^c^	0.63 ± 0.02 ^a^	0.818 ± 0.02 ^a^	185.39 ± 11.92 ^a^	0.505 ± 0.01 ^a^
*F*-value	4.267	37.067	198.528	21.549	11.216	0.295	31.818
*p*-value	0.097	0.002	<0.001	0.006	0.020	0.828	0.003

Different superscript letters (^a–d^) within the same column indicate significant differences according to Duncan’s multiple range test (*p* < 0.05).

**Table 5:** Microstructural parameters of control and pear pomace powder (DP)-substituted muffin samples obtained by image processing.

Sample	Total Air Cell Area	Average Air Cell Size	Air Cell Area (%)	Circularity
C0	43.92 ± 0.14 ^c^	0.164 ± 0.001 ^a^	27.23 ± 0.09 ^c^	0.794 ± 0.002 ^ab^
DP5	39.71 ± 0.57 ^b^	0.104 ± 0.037 ^a^	24.62 ± 0.35 ^b^	0.830 ± 0.013 ^c^
DP10	38.74 ± 0.67 ^b^	0.122 ± 0.014 ^a^	24.02 ± 0.41 ^b^	0.823 ± 0.012 ^bc^
DP20	33.30 ± 0.26 ^a^	0.153 ± 0.079 ^a^	20.65 ± 0.16 ^a^	0.780 ± 0.017 ^a^
*F*-value	177.849	0.772	177.820	7.477
*p*-value	<0.001	0.567	<0.001	0.041

Different superscript letters (^a–c^) within the same column indicate significant differences according to Duncan’s multiple range test (*p* < 0.05).

## Data Availability

The original contributions presented in the study are included in the article/Supplementary Material, further inquiries can be directed to the corresponding author.

## Supplementary Materials

The following supporting information can be downloaded at: https://www.mdpi.com/article/10.3390/foods14223903/s1, Figure S1: Differential scanning calorimetry (DSC) thermograms taro flour; Table S1: Descriptors, scale anchors and descriptor definitions used in the quantitative descriptive analysis; Table S2: Pearson correlation matrix between hardness and porosity; Table S3: Principal Component Analysis (PCA) loadings of sensory attributes for the first four principal components.
