# Supplementary material for: Valorization of Pear Pomace in Taro Gluten-Free Muffins: Composition, Texture, and Sensory Profile
Source: Foods. 2025 Nov 14;14(22):3903. doi: 10.3390/foods14223903 (PMC12651304; doi:10.3390/foods14223903)
Supplement: Supplementary file 1 [file foods-14-03903-s001.zip › SM.pdf]

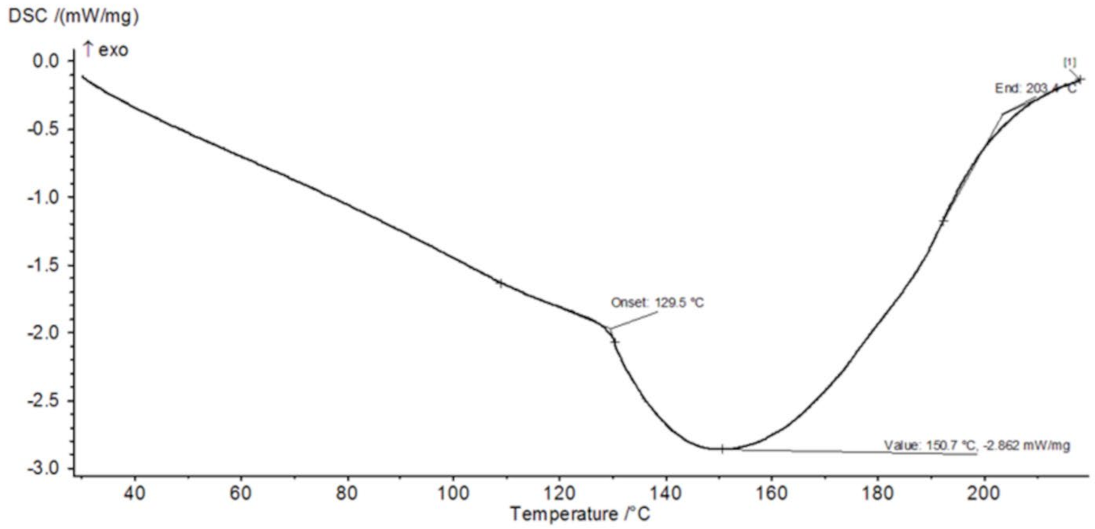

Figure S1. Differential scanning calorimetry (DSC) thermograms taro flour.

Table S1. Descriptors, scale anchors and descriptor definitions used in the quantitative descriptive analysis.

Descriptor	Scale Anchors	Descriptor Definition
**Appearance**		
Crumb porosity	Low (None) - High (Extreme)	Degree of density of crumb related to air bubble formation
Crust color	Low (Light) - High (dark)	Degree of color darkness
Crumb color	Low (Light) - High (dark)	Intensity of crumb browning color
**Texture**		
Hardness	Low (soft) - High (hard)	Force applied to compress muffin via finger
Springiness	Low (inelastic) - High (elastic)	Force applied to compress muffin via finger - Speed of crumb recovery when removing pressure from a finger
**Mouth feel**		
Moistness	Low (Dry) - High (Moist)	Sensed moisture in the mouth from muffin
Oiliness	Low (Not perceived) - High (Intense)	Sensed oiliness after swallowing the muffin
Chewiness	Low (Difficult to chew) - High (easy to chew)	Difficulty when chewing the muffin completely
**Odor**		
Typical Odor	Low (Foreign) - High (Typical)	Typical odor of baked food, consisting of fresh and sweet
Aromatic Odor	Low (Not perceived) - High (Intense)	The intensity of fruit-associated aromatic odor
**Taste**		
Typical Taste	Low (Uncharacteristic) - High (Typical)	Typical taste of baked food, comprising fresh, sweetish and oily; the taste is sweet and aromatic, slightly pungent.
Aromatic Taste	Low (Not perceived) - High (Intense)	The taste is fruity and aromatic, slightly pungent.
After-taste	Low (Disagreeable) - High (Agreeable)	The intensity of the olfactory and/or gustatory sensation that occurs after swallowing

Table S2. Pearson correlation matrix between hardness and porosity.

Variable		Hardness	Porosity
**Hardness**	Pearson Correlation	1	-0.932
	Sig. (2-tailed)		0.001
	N	8	8
**Porosity**	Pearson Correlation	-0.932	1
	Sig. (2-tailed)	0.001	
	N	8	8

Table S3. Principal Component Analysis (PCA) loadings of sensory attributes for the first four principal components.

Sensory Attribute	PC1	PC2	PC3	PC4
Appearance - Crumb Porosity	-0.28	0.1	0.28	0.12
Appearance - Crust Color	0.22	0.74	-0.18	-0.18
Appearance - Crumb Color	0.27	0	0.61	-0.21
Texture - Hardness	0.29	0.05	0.18	0.29
Texture - Springiness	-0.28	-0.17	0.3	-0.25
Mouthfeel - Moistness	-0.29	0.01	0	-0.64
Mouthfeel - Oiliness	0.28	-0.01	0.46	-0.11
Mouthfeel - Chewiness	-0.28	-0.34	0.05	0.22
Odor - Typical Odor	-0.28	0.26	-0.03	-0.22
Odor - Aromatic Odor	0.29	-0.05	0.15	-0.18
Taste - Typical Taste	-0.27	0.33	0.26	0.39
Taste - Aromatic Taste	0.27	-0.34	-0.29	-0.2
Taste - Aftertaste	0.29	-0.05	-0.08	0.1
**Variance Explained (%)**	**91.0**	**6.0**	**3.0**	**0.0**
